# Early Synapse-Specific Alterations of Photoreceptor Mitochondria in the EAE Mouse Model of Multiple Sclerosis

**DOI:** 10.3390/cells14030206

**Published:** 2025-01-30

**Authors:** Dalia R. Ibrahim, Karin Schwarz, Shweta Suiwal, Sofia Maragkou, Frank Schmitz

**Affiliations:** Institute of Anatomy, Department of Neuroanatomy, Medical School Homburg, Saarland University, 66421 Homburg, Germany; karin.schwarz@uks.eu (K.S.); shwetasuiwal@gmail.com (S.S.); sofia.mrg97@gmail.com (S.M.)

**Keywords:** retina, ribbon synapse, multiple sclerosis, experimental autoimmune encephalomyelitis (EAE), mitochondria, MIC60, DRP1, synaptic mitochondria, extra-synaptic mitochondria

## Abstract

Multiple sclerosis (MS) is an inflammatory autoimmune disease of the central nervous system (CNS) linked to many neurological disabilities. The visual system is frequently impaired in MS. In previous studies, we observed early malfunctions of rod photoreceptor ribbon synapses in the EAE mouse model of MS that included alterations in synaptic vesicle cycling and disturbances of presynaptic Ca^2+^ homeostasis. Since these presynaptic events are highly energy-demanding, we analyzed whether synaptic mitochondria, which play a major role in synaptic energy metabolism, might be involved at that early stage. Rod photoreceptor presynaptic terminals contain a single large mitochondrion next to the synaptic ribbon. In the present study, we analyzed the expression of functionally relevant mitochondrial proteins (MIC60, ATP5B, COX1, PINK1, DRP1) by high-resolution qualitative and quantitative immunofluorescence microscopy, immunogold electron microscopy and quantitative Western blot experiments. We observed a decreased expression of many functionally relevant proteins in the synaptic mitochondria of EAE photoreceptors at an early stage, suggesting that early mitochondrial dysfunctions play an important role in the early synapse pathology. Interestingly, mitochondria in presynaptic photoreceptor terminals were strongly compromised in early EAE, whereas extra-synaptic mitochondria in photoreceptor inner segments remained unchanged, demonstrating a functional heterogeneity of photoreceptor mitochondria.

## 1. Introduction

Multiple sclerosis (MS) is a neuro-inflammatory autoimmune disease of the central nervous system (CNS) linked to a plethora of neurological disabilities [1,2,3,4,5]. Nearly 3 million people, with rising prevalence, are affected by MS worldwide [6,7]. Young female adults in particular suffer from the disease. MS is typically considered a white matter disease of the CNS, characterized by the demyelination of axonal fibre tracts. Recently, it became clear that the grey matter is also affected in MS, even at an early disease stage [8,9,10,11,12,13,14]. Within the grey matter, neurodegeneration and changes in synapse structure and function were identified [5,13,14,15,16,17].

Multiple sclerosis frequently affects the visual system [18,19,20]. Similar to the brain, alterations in the visual system in MS patients or animal models of MS have been observed in the grey and white matter. The inflammation and demyelination of the optic nerve, a white matter region that physiologically contains the myelinated axons of retinal ganglion cells, are typical symptoms in MS [21,22,23,24]. The retina, which belongs to the grey matter of the visual system, is also targeted by the auto-aggressive immune system in MS. Alterations in photoreceptor synapses and bipolar cell synapses of the retina have been observed in the EAE (Experimental Autoimmune Encephalomyelitis) mouse model of MS [25,26,27,28,29]. Of note, photoreceptor synapse dysfunctions occurred during an early pre-clinical stage in EAE mouse retinas before obvious signs of demyelination in the optic nerve, suggesting that synapse dysfunctions are not secondary to demyelination but independent, early events of the disease [25]. In the present study, we aimed to obtain further insights and a better understanding on EAE-induced early alterations in retinal photoreceptor synapses.

Photoreceptor synapses and retinal bipolar cell synapses are continuously active glutamatergic ribbon synapses [30,31]. These synapses possess structural, molecular, and functional specializations to foster continuous synaptic transmission. The synaptic ribbon is the most prominent morphological specialization of ribbon synapses that guides synaptic vesicles to the active zone where exocytosis occurs. It mainly consists of the protein RIBEYE [32]. Exocytosis and synaptic vesicle cycling at ribbon synapses are controlled by presynaptic Ca^2+^, and the continuous vesicle cycling requires efficient Ca^2+^ extrusion mechanisms [30]. Ca^2+^ extrusion mechanisms and the continuous activity of synaptic vesicle cycle are highly energy-demanding [33] and require a strong ATP-generating system in the presynaptic terminal [34,35]. A powerful and highly dynamic energy-generating system is provided by synaptic mitochondria [34,35]. Mitochondrial function at presynaptic terminals is regulated by transient increases in presynaptic Ca^2+^, which adjusts mitochondrial function and energy production to synaptic activity [35,36]. Chronic Ca^2+^ overload can damage mitochondria [12,37,38,39,40].

Mitochondrial dysfunctions have been suggested to have an important impact on the pathogenesis of MS [41,42]. Many of the supporting data have been obtained from MS post-mortem tissue with the disease already present at an advanced stage (e.g., [39,43,44,45,46]), but also data from the EAE model of MS pointed to a role of mitochondria in MS pathogenesis [47,48].

In the current study, we applied the EAE mouse model of MS to analyze whether synaptic mitochondria could play a role in early photoreceptor synapse dysfunctions in pre-clinical EAE. We focused on mitochondria in photoreceptor synapses because these synapses were strongly affected in early pre-clinical EAE [25,26,27]. The mouse retina contains mostly rod photoreceptors that possess a characteristic morphology with a bell-shaped presynaptic terminal [30]. The bell-shaped presynaptic terminal surrounds the postsynaptic cavity that harbours slender postsynaptic dendrites from horizontal and bipolar cells [49,50,51,52]. In the vascularized retina of mice, rod photoreceptor presynaptic terminals possess a single large mitochondrion close to the synaptic ribbon for mitochondrial ATP production [49,50,52]. Due to its very large size, this synaptic mitochondrion is particularly accessible to morphological and functional investigations. On the postsynaptic side, the thin, slender postsynaptic dendrites within the postsynaptic cavity typically do not contain mitochondria [49,50,52]. Therefore, mitochondria in close vicinity to the synaptic ribbon in the OPL can be largely assigned to presynaptic mitochondria of photoreceptor terminals. The mitochondria in the dendritic processes of horizontal and bipolar cells are predominantly located outside of the postsynaptic cavity of the photoreceptor synapse in the neuropil sub-layer of the OPL close to the inner nuclear layer [26,49,50,52]. We found alterations in functionally relevant mitochondrial proteins (MIC60, ATP5B, COX1, Pink1, DRP1; see below) in photoreceptor synapses already early in pre-clinical EAE, suggesting that early mitochondrial dysfunctions in the presynaptic terminals of glutamatergic photoreceptor synapses could play an important role in the previously observed early synaptic dysfunctions in EAE.

## 2. Materials and Methods

### 2.1. Mice

All animal procedures were monitored and approved by the responsible animal authorities (Landesamt für Verbraucherschutz; GB 3; 66115 Saarbrücken, Germany). Animal experiments were performed under animal experimentation permit GB3-2.4.2.2-25-2020 issued by the Landesamt für Verbraucherschutz, GB 3, 66115 Saarbrücken, Germany. As in previous studies [25,26,27,53], female, non-pregnant C57BL/6J mice were used for EAE experiments for the sake of comparability with previous studies and to minimize potential variability. Furthermore, gender-specific differences have been observed in the EAE animal model of MS that could be related to gender-specific aspects of MS also in humans, e.g., the higher incidence of MS in female humans [54,55,56,57,58,59,60]. But it is possible to induce EAE also in male mice [61]. In the present study, only female mice older than 10 weeks with a body weight between 20 g and 25 g were used for EAE induction to exclude possible gender-specific effects. Mice were kept on a 12-h light-dark cycle and provided with standard food and water ad libitum.

### 2.2. Antibodies

Primary Antibodies (Table 1).

Secondary antibodies (Table 2)

### 2.3. Methods

#### 2.3.1. Induction of EAE in C57BL/6J Mice

In the present study, we used the Experimental Autoimmune Encephalomyelitis (EAE) model of multiple sclerosis [54,55,56,57,58,59,60,61]. For EAE induction (including control injections; see below), only female animals were used as in our previous studies [25,26,27,53,79]. EAE inductions in female C57BL/6J mice were performed as previously described [25,26,27,53]. Mice were randomly allocated to the two different groups, i.e., experimental EAE group or negative control group. For EAE inductions, mice were injected subcutaneously into axilla and groin with a total of 200 µg of MOG_35–55_ peptide (MEVGWYRSPFSRV VHLYRNGK). The peptide was thoroughly suspended in complete Freund’s adjuvant (CFA). Mice that were injected with the same volume of CFA only served as negative controls. On the same day, 1–2 h after MOG/CFA or CFA-only injection, and on the next day, both EAE and control mice were injected intraperitoneally with pertussis toxin (PTX) (List Biological Laboratories/Biozol (Eching, Germany #181). In total, 200 ng PTX was used for each injection, as previously described [25,26,27,53]. After injection, MOG/CFA-injected EAE mice and CFA-injected control mice were housed in the same cage under identical conditions. EAE induction was performed by an experimenter that was not involved in subsequent immunolabelling experiments. EAE induction with MOG/CFA was always performed in parallel to CFA control injections within the same experiment. All experiments were performed in a blinded manner, i.e., the experimenter did not know whether they were dealing with samples from MOG/CFA-injected EAE mice or samples from CFA-injected control mice. Retinas of both groups (experimental and control) were isolated on day 9 after injection, as in previous studies [25,26,27,53], for the sake of comparability. Day 9 after injection is within the pre-clinical phase of EAE [54,55,56,57,58,59,60,61], in which no scorable clinically symptoms, as defined by the standard EAE score sheets [54,55,56,57,58,59,60,61] (e.g., limb paralysis), are present. These scorable clinically symptoms of the standard EAE score sheets [54,55,56,57,58,59,60,61] typically do not appear before day 10 after injection. At the beginning of the clinical phase, these scorable clinical symptoms start to develop and are recorded in the standard EAE score sheets [54,55,56,57,58,59,60,61]. Consistent with this time course, we did not observe any EAE sheet-scorable clinical symptoms in our EAE mice sacrificed on day 9 after injection. This early time point (day 9 after injection) was chosen on purpose for our analyses in order to minimize the possibility that the synaptic alterations in the retina and the underlying mechanisms could be secondary to other pathologies, e.g., optic neuritis/demyelination. Sample size, experimental power, and statistical analyses were performed as described below.

#### 2.3.2. Immunofluorescence Microscopy

##### Embedding of Retinal Samples in Epon for Immunolabelling of 0.5 µm Thin Resin Sections

The eyes were enucleated, and the posterior eye - cups containing the retina were dissected from MOG/CFA-injected EAE mice and CFA-injected control mice within 5 min post-mortem, as previously described [25,26,27,53]. Samples were flash-frozen in isopentane-liquid nitrogen and freeze-dried in a liquid nitrogen-cooled chamber for ≈48 h, as previously described [25,26,27,53,80,81,82,83,84]. After equilibration to room temperature, the samples were infiltrated with Epon resin (overnight at 28 °C in an overhead rotator at 2 rpm). Epon-infiltrated samples were polymerized at 60 °C for 48 h [25,26,27,62,85]. This is a paraformaldehyde-free procedure that provides a high signal-to noise ratio in immunolabelling analyses, yielding strong immunofluorescence signals [25,26,27,53].

From the tissue blocks, 0.5 µm thin (semi-thin) sections were prepared with a Reichert ultramicrotome UltraCut E (Reichert-Jung, Nußloch, Germany) using a diamond knife (Diatome AG; Nidau, Switzerland) and transferred to glass coverslips. The coverslips with the transferred resin sections were heated for ≈30 min on a heating pad (60 °C) for the firm attachment of the sections to the glass surface and subsequently stored at room temperature (RT) until use. Experimental and control sections were processed for subsequent Epon removal in the same cuvette in a blinded manner. Epon removal from the retinal resin sections was performed as previously described [25,26,27,80,81,82,83,84]. For Epon removal, the sections were subsequently incubated with 30% Na-Methylate (in methanol) solution (Sigma/Merck, #8.18194.0100), xylene/methanol 1:1 (*v*/*v*), acetone (2x), H_2_O, and phosphate-buffered saline (PBS) (10–13 min each step). Afterward, sections were processed for immunolabelling as described below.

##### Immunolabelling of 0.5 µm Thin Resin Sections from the Mouse Retina

We used 0.5 µm thin (semi-thin) sections of the retina for immunolabelling, as previously described [25,26,27,80,81,82,83,84]. These thin sections provide a particularly high resolution for morphological analyses and a high signal-to-noise ratio with low background [25,80,81,82,83,86]. For double - immunolabelling, the two primary antibodies from two different species were applied simultaneously, in the optimized concentrations presented in Table 1. Incubation in the primary antibody solutions was performed overnight at 4 °C. On the next day, sections were rinsed several times with PBS to remove unbound antibodies and incubated with the respective secondary antibodies at the dilutions summarized in Table 2 (2 hr incubation, RT). After incubation with the secondary antibodies, sections were washed several times with PBS and embedded in N-propyl gallate (NPG) anti-fade, as previously described [25,26,27,80,81,82,83,84]. Negative control incubations were performed, in which the primary antibodies were omitted with the remaining immunolabelling procedure remaining completely the same. In each experiment, at least 5 independent pairs of MOG/CFA-injected EAE mice and CFA-injected control mice were used for immunolabeling, as mentioned in the specific experiments.

##### Confocal Microscopy

An A1R confocal microscope (Nikon, Düsseldorf, Germany) under the control of the NIS Elements software (AR 3.2, 64bit) was used for imaging, as previously described [25,26,27,54,80,81,82,83,84]. Images were acquired with a 60×/1.40 N.A. oil objective [25,26,27,54,80,81,82,83,84]. Images were acquired blindly, with the experimenter not knowing whether they were imaging an experimental or control section. For the quantification of immunofluorescence signals, identical imaging conditions were applied using the “re-use camera settings” of the NIS Elements software (NIS Elements AR 3.2, 64bit).

##### Quantification of Immunosignals in the Outer Plexiform Layer (OPL)

Immunosignals in OPL were quantified through the subsequent procedure. Confocal images obtained from the A1R confocal microscope were exported as tiff files. The area of interest was determined by drawing a rectangular region of interest (ROI) along the entire outer plexiform layer (OPL) using NIH ImageJ software (Fiji, version 1.48F; [87,88]). The rectangular ROI covered the entire OPL that was defined using the RIBEYE immunosignals. The RIBEYE immunosignals in the OPL served as a landmark to determine the border of the OPL and thus define the OPL ROI. The split channel choice was used to apply the same rectangular ROI on the protein of interest in the OPL. The identical ROIs were used for CFA and MOG/CFA samples by selecting the same ROI in the Analyse-tools-ROI manager in ImageJ (Fiji version 1.48F; [87,88]). Each experiment was repeated at least 5 times in a blinded manner, with the experimenter not knowing whether the sample was from a CFA- or MOG/CFA-injected animal. The integrated density for all images was analyzed for immunofluorescence analysis. Then, the average of the integrated density values of the CFA images was calculated and compared to the average integrated density of MOG/CFA images in each experiment. The average integrated density values for MOG/CFA were normalized to the average of CFA values (in %). Statistical significance was determined in GraphPad 9.5.1 Prism, as described below [25,26,27,62,81,84].

##### Analyses of MIC60 Immunosignals from Individual Synaptic Mitochondria in the OPL

For MIC60/Mitofilin we also quantified the fluorescence intensity of single synaptic mitochondria in the outer plexiform layer (OPL) of MOG/CFA-injected EAE mice and CFA-injected control mice. Highly magnified confocal images were obtained from the A1R confocal microscope and exported as tiff files. Fluorescence intensities were determined as described above for the analyses of fluorescence signal intensities in the OPL. In these experiments, the ROIs were placed only on the individual MIC60/Mitofilin puncta (i.e., individual mitochondria) in close vicinity to the synaptic ribbons. For the individual analyses of fluorescence signal intensities of single synaptic MIC60/Mitofilin-labelled mitochondria close to the synaptic ribbons, the freehand tool of NIH ImageJ software (Fiji, version 1.48F; [87,88]) was used to outline the border of the single synaptic mitochondria. The immunolabelled individual synaptic mitochondria ROIs defined in this way were analyzed for integrated density in a blinded manner, as described above.

#### 2.3.3. Transmission Electron Microscopy (TEM) of Mitochondria of Rod Photoreceptor Synapses

Retinas from CFA- and MOG/CFA-injected animals were processed for conventional transmission electron microscopy (TEM), largely as previously described [27,89]. For TEM embedding, eyes were enucleated, and retinas dissected within 5 min post-mortem. Samples were immediately fixed with 2% (*w*/*v*) freshly depolymerized paraformaldehyde (PFA) and 1.25% (v/v) glutaraldehyde in PBS (all final concentrations) for 12 h each at 4 °C at constant mild agitation. After several washes with PBS and 100 mM cacodylate buffer, retinas were treated with 1% (*w*/*v*) OsO_4_/1.5% potassium ferricyanide in 100 mM cacodylate buffer, pH 7.4 (2 h at 4 °C with gentle agitation). Following several washes with H_2_O and 50 mM Na^+^-maleate buffer (pH 5.0), samples were contrasted with 2% uranyl acetate in 50 mM Na^+^-maleate buffer (pH 5.0, 3 h at 4 °C with gentle agitation). After contrasting, samples were dehydrated by increasing concentrations of ethanol (30%, 50%, 70%, 80%, 90%, 99%; 20 min each step at RT) and acetone (20 min, RT). Next, samples were gradually infiltrated with Epon, using acetone/Epon mixtures (3:1, 1:1, 1:3 (*v*/*v*), 1 h each step, at RT). Then, samples were infiltrated with pure Epon resin overnight at RT with mild agitation using an overhead rotator at 2 rpm. Epon-infiltrated samples were polymerized at 60 °C for ≈48 h. Ultrathin sections (≈70 nm thin) were sectioned from these samples with an UltraCut E ultramicrotome (Reichert-Jung, Nußloch, Germany).

Ultrastructural analyses were performed with a digital transmission electron microscope (Tecnai 12 Biotwin; FEI, Eindhoven, The Netherlands) equipped with a Megaview III digital camera (Gatan, Weiterstadt, Germany) that was controlled by the iTEM acquisition software (Olympus, Hamburg, Germany). Rod synapses and mitochondria were identified by their characteristic shape. For the determination of synapse and mitochondria density, a magnification of 8200X was used both for MOG/CFA and CFA samples. The number of rod synapses and synaptic mitochondria were normalized to 100 µm of OPL length (along the ONL). The scale bar on the EM images was used for the normalization. Mitochondria were also evaluated at a higher magnification to observe potential differences in the ultrastructural organization of mitochondria in EAE mice versus control-injected mice.

##### Measurement of the Diameter of the Synaptic Mitochondrion in the OPL of Rod Photoreceptor Synapses Using TEM Images

For a semi-quantitative evaluation of the diameter of the presynaptic mitochondrion in rod photoreceptor synapses, transmission electron micrographs from MOG/CFA-injected EAE samples and CFA-injected control samples at day 9 after injection were acquired blindly at a primary magnification of 8200X. The option of straight freehand lines in NIH ImageJ software (Fiji, version 1.48F; [87,88]) was used to draw and measure the diameter of each mitochondrion by passing a line from two random points of the outer mitochondrial membrane through the centre of the mitochondrion. The values were analyzed with Microsoft Excel, and the arithmetic mean values were calculated. The diameter values were analyzed by an experimenter who was not aware whether the sample was from a CFA- or MOG/CFA-injected sample. The scale bar of the TEM images was used for the calibration/transformation of the measurements into absolute numbers.

#### 2.3.4. Post-Embedding Immunogold Labelling

##### Embedding of Retinas in LR Gold Resin for Immunogold Electron Microscopy

The embedding of retinas in LR Gold resin for post-embedding immunogold labelling was performed as previously described [32,85]. Briefly, freshly isolated retinas were fixed with 2% freshly depolymerized paraformaldehyde (PFA) and 0.1% glutaraldehyde (GA) in PBS, pH 7.4, for 12 h at 4 °C. Next, samples were washed with PBS and treated with tannic acid (0.1%, w/v in PBS) for 1 h at 4 °C. After several washes with H_2_O and 50 mM maleate buffer, samples were contrasted with filtered 2% uranyl acetate in H_2_O for 2 h at 4 °C. Subsequently, probes were dehydrated using an ascending ethanol concentration at 4 °C (30% ethanol) to −20 °C (all other ethanol steps) to minimize the extraction of lipids. Dehydration was performed in steps of 30%, 50%, 70%, 80%, 90%, and 99% pre-cooled ethanol (30 min each). Afterward, samples were infiltrated with London Resin (LR) Gold (Electron Microscopic Sciences). The LR Gold resin solution was changed thrice and finally replaced with LR Gold containing 0.1% benzil as a polymerization catalyst. Polymerization was performed with UV light at −20 °C for ≈48 h. From the polymerized tissue blocks, ≈70 nm thick sections were cut with an ultramicrotome (Reichert-Jung) and collected on 100 mesh gold grids.

##### Post-Embedding Immunogold Labelling of Ultrathin Sections

MIC60/Mitofilin immunogold puncta density on rod photoreceptor mitochondria was determined by post-embedding immunogold electron microscopy. For immunolabelling, grids were first incubated with 0.5% bovine serum albumin (BSA) in PBS (blocking buffer) for 45 min at RT to block unspecific binding sites. Then, the grids were incubated in the indicated primary antibodies in the dilutions given in Table 1 (ON, 4 °C). In the negative control incubation, primary antibody was omitted. On the next day, the grids were washed with PBS 4 times (5 min each) to remove unbound antibodies. Next, the grids were incubated with goat anti-rabbit secondary antibody conjugated to 5 nm gold particles (1:100 in blocking buffer, 1 h at RT). Afterwards, section were washed again with PBS several times before the fixation of the immune complexes with 2.5% glutaraldehyde in PBS (15 min, RT). This step was followed by washing with H_2_O four times at RT and incubation with 2% uranyl acetate (UA) for 15–18 min [32,85]. Lastly, sections were rinsed with H_2_O again and left to dry. Images were acquired with an Tecnai Biotwin12 digital transmission electron microscope in a blinded manner, i.e., without the experimenter knowing which sample is from CFA- or MOG/CFA-injected mice.

##### Quantification of MIC60/Mitofilin Immunogold Puncta on Presynaptic Mitochondria of Rod Photoreceptor Synapses

MIC60/Mitofilin immunogold puncta were analyzed and quantified on TEM (transmission electron microscope) images obtained at a primary magnification of 43,000X. The immunogold puncta per synaptic mitochondrion were quantified. The area of each single mitochondrion was determined by taking advantage of the freehand tool in ImageJ (Fiji, version 1.48F; [87,88]). The scale bar of the TEM images was used for the normalization of the area of the mitochondria. All immunogold particles within the mitochondrial area were manually counted using NIH ImageJ (Fiji, version 1.48F; [87,88]). Data from MOG/CFA-injected EAE samples and CFA-injected control samples were collected in a blinded manner. Mean values and standard errors of the mean (S.E.M.) were analyzed using Microsoft Excel; statistical analyses were performed with GraphPad prism 9.5.1. The arithmetic mean of the CFA values was set to 100%, and the values from the MOG/CFA samples were related to it. MIC60 immunogold puncta on presynaptic mitochondria were also analyzed as the number of immunogold puncta per area of presynaptic mitochondria (MIC60 immunogold labelling density on presynaptic mitochondria in rod photoreceptor synapses). For this purpose, immunogold puncta were manually counted in a blinded manner and related to the area of the presynaptic mitochondrion. The area of the mitochondrion was determined with the help of the scale bar of the respective TEM images.

#### 2.3.5. Western Blot Analyses of CFA and MOG/CFA Retina Samples and Quantification of Western Blot Bands with the LI-COR System

Retinas were isolated from enucleated eyes of CFA- or MOG/CFA-injected mice within 5 min post-mortem, as previously described [26,27,62]. Isolated retinas were dissolved in hot sodium dodecylsulfate (SDS) Laemmli sample buffer (50 μL for each retina) followed by heating at 96 °C (10 min with gentle agitation). For protein quantification, we used the amido black protocol that also works with samples dissolved in Laemmli sample buffer [90]. Eight pairs of retinas from CFA-injected control mice and MOG/CFA-injected EAE mice obtained at day 9 after immunization were used for the analyses. For comparison between CFA and MOG/CFA, 30 µg of the protein lysate was analyzed by Western blot with the indicated antibodies (Table 1). After electrotransfer, nitrocellulose membranes were washed 4 times (10 min) with PBS before blocking unspecific binding sites by incubation with 5% skimmed milk in PBS at RT on a shaker. Primary antibody solutions (MIC60/Mitofilin, DRP1, Actin) were applied overnight at 4 °C at the dilutions given in Table 1. Actin served as a loading control. After incubations in the primary antibodies and several washes with PBS, the binding of the primary antibodies was visualized with donkey anti-Rabbit IR Dye 800cw or donkey anti-mouse IR Dye 680LT (1:5000 dilution in 3% milk for 2 h at RT). After several washes with PBS, the binding of the fluorescent secondary antibody was visualized and quantified with a LI-COR Odyssey Imaging system. The Thermo Scientific PageRuler prestained protein ladder (ThermoScientific, Dreieich, Germany, MAN0011772) was used as a protein marker in the experiments. Please note that the nitrocellulose membranes were incubated sequentially with the indicated primary (and secondary) antibodies without stripping because the analyzed proteins could be clearly separated by their characteristic running position in Western blots. We omitted the stripping of the antibodies from the nitrocellulose membranes because the antibodies tightly bound to their antigen immobilized on the nitrocellulose membrane and could not be reliably removed with standard procedures, e.g., [62], without compromising the nitrocellulose membrane for further rounds of incubations with antibodies. Please also note that the LI-COR Odyssey system allows for multiplexing, i.e., primary antibodies from two different species can be detected simultaneously in different detection channels using the respective secondary antibodies conjugated to different fluorophores (Table 2).

##### Quantification of Western Blot Bands

The quantification of single Western blot bands was performed with Image Studio Software (Image Studio Lite 5.2 software; LI-COR), as previously described [26,62]. The area of interest was determined by drawing a rectangle to the specific band using the add rectangle tool. The exact signal intensity values were exported to Microsoft Excel. The Western blot signals of MIC60/Mitofilin and DRP1 were normalized to the actin signal of the same respective lane. The expression of actin was not altered in EAE retinas versus control retinas at day 9 after injection [25]. The unaltered expression of actin in EAE vs. control retinas at day 9 after injection was confirmed in the present study (*p* = 0.8884; unpaired Student’s *t*-test, i.e., non-significant). In each separate experiment, the MOG/CFA value was normalized to the respective CFA reference value. The CFA value was set to 100% in each experiment. Eight littermate pairs were used in this experiment (N = 8). The normalized data were analyzed for normal or non-normal distribution with the Shapiro–Wilk test using GraphPad Prism 9.5.1. Depending on the data distribution (normal or non-normal), the data were analyzed for statistical significance with a one-sample Student‘s *t*-test for non-equal variance (in case of normally distributed data) or with a one-sample Wilcoxon test (in case of non-normally distributed data) implemented in GraphPad Prism 9.5.1). The hypothetical mean was set to 100. *p* < 0.05 was considered a significant difference. GraphPad Prims 9.5.1 was used to plot column diagrams that show the arithmetic means ± S.E.M.s. In the column plots, the individual values from the respective experiments were also depicted.

#### 2.3.6. Statistical Analyses

Based on a priori sample size estimations (α ≤ 0.05; effect size Cohen’s d = 0.6; power = 0.8) with G*Power Version 3.1.9.6 [91,92] and our previous experience, a total of 3-5 independent immunizations were performed. Each set of immunizations was composed of CFA-injected control animals and MOG/CFA-injected experimental animals. The actual values for effect sizes and statistical power were calculated through post hoc analyses with G*Power Version 3.1.9.6, as given in the figure legends of their respective experiments.

GraphPad Prism software (version 9.5.1) was used for the statistical analyses of the data. First, we analyzed whether the data from the independent experiments could be pooled. For this purpose, we determined whether the reference data of the different individual experiments from the CFA control group differed significantly from each other or not. Shapiro–Wilk tests were used to analyze whether data were normally distributed or not. For normally distributed data, ANOVAs analyses (with Tukey’s correction for multiple comparisons) were performed to test for potential statistical differences among the data from the different independent experiments of the control group (CFA-injected animals). If data were not normally distributed, Kruskal–Wallis analyses (with Dunn correction for multiple comparisons) were performed. If no statistical differences were observed in the CFA control groups of the different experiments (experimental repeats), which was the case in our experiments, data were pooled within each group, and the pooled data were compared with each other (e.g., CFA vs. MOG/CFA). If the pooled data were normally distributed as judged by Shapiro–Wilk analyses, the parametric unpaired Student’s *t*-test was used for single comparisons. If data were not normally distributed, the non-parametric Mann–Whitney U-test was used for single comparisons. Differences were considered statistically significant with a *p*-value smaller than 0.05 (*p* < 0.05). Finally, column graphs were plotted to demonstrate the arithmetic means of the data together with the standard errors of the mean (S.E.M.). The data were also plotted as box-and-whisker plots to show the individual data distribution with Origin 2019b.

## 3. Results

In the current study, we analyzed synaptic mitochondria of photoreceptor synapses in the outer plexiform layer (OPL) of the retina for morphological pathologies on day 9 after injection in the EAE mouse model of multiple sclerosis. Previous analyses demonstrated photoreceptor synapse dysfunctions at this early time point in the pre-clinical phase of EAE [25,26,27,53]. In the present study, we wanted to further analyze the underlying molecular mechanisms and a possible involvement of synaptic mitochondria.

**Figure 1:**
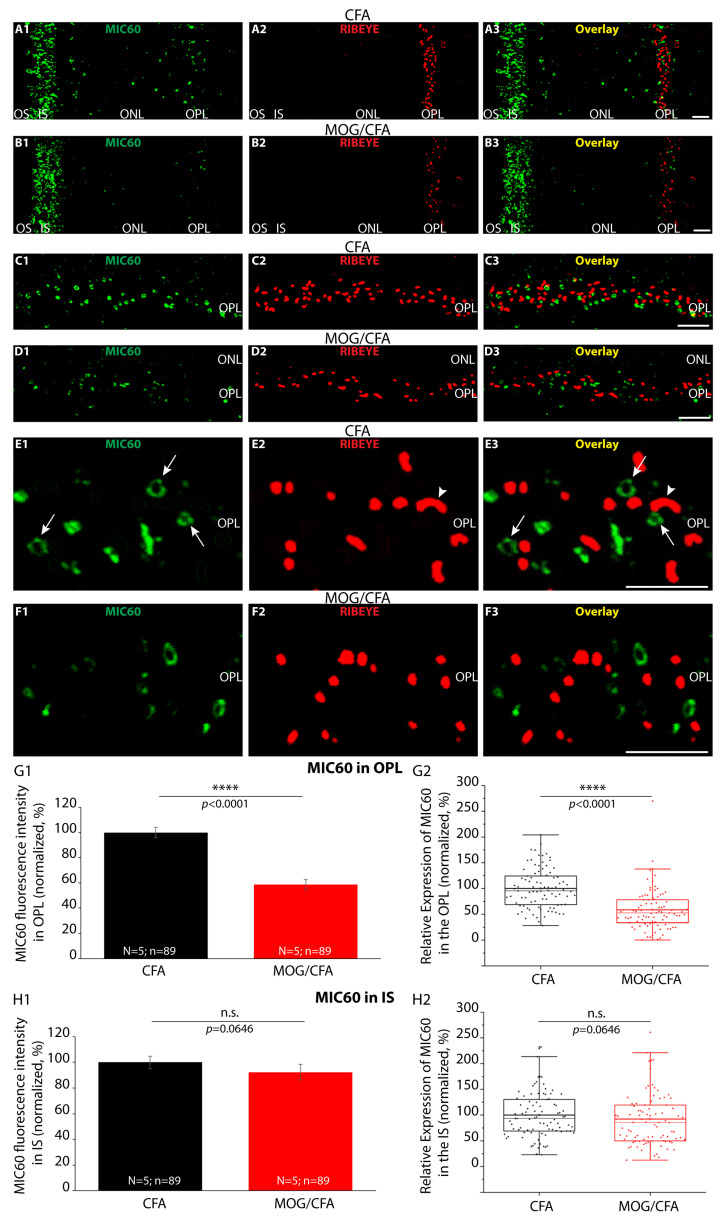
Decreased MIC60 immunosignals in the OPL of MOG/CFA-injected mice in comparison to CFA-injected mice at day 9 post-injection. The 0.5 µm- thin retina sections from MOG/CFA-injected EAE mice (**B1**–**B3**,**D1**–**D3**,**F1**–**F3**) and CFA-injected control mice (**A1**–**A3**,**C1**–**C3**,**E1**–**E3**) double-immunolabelled with rabbit polyclonal MIC60 antibody (green channel) and mouse monoclonal RIBEYE antibody (red channel). Signals from the green (**A1**,**B1**,**C1**,**D1**,**E1**,**F1**) channels and red channels (**A2**,**B2**,**C2**,**D2**,**E2**,**F2**) were merged in (**A3**,**B3**,**C3**,**D3**,**E3**,**F3**). (**A1**–**B3**) Representative low-magnification images. Low-magnification micrographs show MIC60-labelled mitochondria in the OPL and in the photoreceptor inner segments in the same section. (**C1**–**D3**) MIC60-positive mitochondria in the OPL close to the synaptic ribbon at intermediate magnification. (**E1**–**F3**) High-magnification confocal images of single immunolabelled synaptic mitochondria in the OPL. Ring-like MIC60 immunosignals of individual presynaptic mitochondria are visible close to the synaptic ribbons (arrows). Ribbons appear as single horseshoe-labelled structures (arrowheads). (**G1**,**G2**,**H1**,**H2**) MIC60 immunosignals from the OPL and IS were quantified as integrated density. (**G1**,**H1**) Bar graphs depict normalized arithmetic means ± S.E.Ms. MOG/CFA integrated density immunofluorescence values were normalized to the mean of the CFA control values (set to 100%). In (**G2**,**H2**), the same data as in (**G1**,**H1**) are depicted as box-and-whisker plots to show the individual datapoints and their distribution. (**G2**,**H2**) Boxes represent 25th–75th percentiles of the data points, mean and median values are indicated by thin and thick horizontal lines, respectively, and whiskers are equal to 1.5 times the interquartile range (IQR). Mann–Whitney U-test was conducted to determine if there were significant differences in MIC60 immunosignals in the OPL between CFA and MOG/CFA groups. The distributions significantly differed between both groups (effect size d = 1.0751; power = 0.9999). MIC60 immunosignals in the inner segments did not differ between both groups (effect size d = 0.1497; power: 0.1517). *p*-values < 0.05 were considered statistically significant. Abbreviations: CFA, complete Freund’s adjuvant; MOG, myelin oligodendrocyte glycoprotein; EAE, experimental autoimmune encephalomyelitis; IF, immunofluorescence; OS, outer segment; IS, inner segment; OPL, outer plexiform layer; ONL, outer nuclear layer; S.E.M., standard error of the mean; N = number of independent experiments; n = number of images analyzed from retinal sections; n.s., non-significant; ****, *p* < 0.0001. Scale bars: 5 μm.

First, we analyzed the mitochondrial protein MIC60/Mitofilin for alterations in the presynaptic mitochondria of photoreceptor synapses. Different names have been assigned to MIC60, including Mitofilin and others. For simplicity, we only use the name MIC60 in the following text. MIC60 is a central component of the mitochondrial contact site and cristae organizing system (MICOS), complex and crucial for mitochondrial function and cristae organization [93,94,95,96,97,98,99]. We observed a strong decrease in MIC60 immunofluorescence signals in the OPL of retinas from MOG/CFA-injected samples in comparison to retinas from CFA-injected control mice at 9 days after injection (Figure 1). The RIBEYE immunosignals, which show the presence of presynaptic ribbons in photoreceptor synapses in the OPL [25,26,27,32,53], were used to determine the location of presynaptic mitochondria in the OPL close to the synaptic ribbon in these and all following experiments. Higher magnification confocal analyses of MIC60 immunolabelled mitochondria in the OPL revealed ring-like immunolabelled structures close to the synaptic ribbon (arrows in Figure 1E1–E3). These ring-like structures reflect the known enrichment of MIC60 in the inner mitochondrial membrane and demonstrate the high resolution of the applied immunolabelling method.

These findings demonstrate that MIC60, one core component of the MICOS complex, is reduced in presynaptic mitochondria of photoreceptor synapses in the OPL of EAE retinas. Our subsequent analyses demonstrated that the reduced expression of MIC60 is specific for synaptic mitochondria and does not occur in the inner segments (IS) of photoreceptors. The inner segments of photoreceptors also contain many mitochondria (e.g., [100,101,102]) and consequently showed a strong MIC60 immunofluorescence signal (Figure 1). In contrast to the MIC60 signal in synaptic mitochondria, the MIC60 immunosignals in the mitochondria of photoreceptor inner segments were qualitatively and quantitatively not statistically different between the retinas of CFA- and MOG/CFA-injected animals (Figure 1A1–A3,B1–B3,H1). These data point to a functional heterogeneity of photoreceptor mitochondria and a differential sensitivity to EAE induction. The higher sensitivity of synaptic mitochondria to EAE induction could be based on a disturbed presynaptic Ca^2+^ homeostasis in the presynaptic photoreceptor terminals in EAE [26]; see the Discussion.

**Figure 2:**
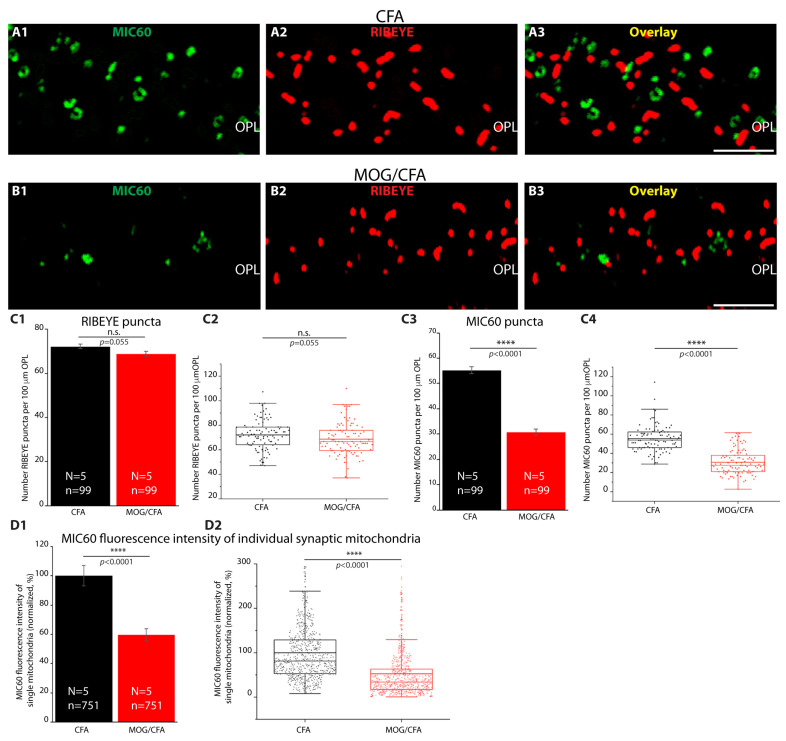
Decreased expression of MIC60 in individual mitochondria of rod photoreceptor synapses in MOG/CFA-injected EAE mice at day 9 after immunization. The 0.5 µm thin retina sections immunolabelled with rabbit polyclonal MIC60 antibody (**A1**,**B1**) and with mouse monoclonal RIBEYE antibody (**A2**,**B2**). Overlay images are shown in (**A3**,**B3**). Highly magnified confocal images of the immunolabelled OPL (A1-B3) were used for the quantification of RIBEYE puncta (**C1**,**C2**) and MIC60 puncta (**C3**,**C4**). (**D1**,**D2**) Quantification of MIC60 fluorescence intensities of each single MIC60-positive synaptic mitochondrion close to rod synaptic ribbons, measured as integrated density. (**C1**,**C3**,**D1**) Bar graphs depict normalized arithmetic means ± S.E.M. In (**C2**,**C4**,**D2**), the individual data from (**C1**,**C3**,**D1**) are depicted as box-and-whisker plots. Boxes represent 25th–75th percentiles; the mean and median values are indicated by thick and thin horizontal lines, respectively. Whiskers are equal to 1.5 times the interquartile range (IQR). Unpaired Student’s *t*-test was conducted to determine if there were significant differences in RIBEYE puncta in the OPL between CFA and MOG/CFA groups. The distributions did not differ significantly between both groups (effect size d = 0.2738, power = 0.4827). To determine differences in MIC60 puncta between both groups the Mann–Whitney U-test was conducted and showed significant difference between both groups (effect size d = 1.8338; power =1.0). Differences in MIC60 expression in individual mitochondria were evaluated with Mann–Whitney U-test. Analysis revealed significant differences between the CFA and MOG/CFA groups (effect size d = 0.7443; power = 1.0). Data were considered statistically different with *p* < 0.05. Abbreviations: EAE, experimental autoimmune encephalomyelitis; CFA, complete Freund’s adjuvant; MOG, myelin oligodendrocyte glycoprotein; OPL, outer plexiform layer; S.E.M., standard error of the mean; N = number of independent experiments; n = number of images analyzed from retinal sections; ****, *p* < 0.0001; n.s., non-significant. Scale bars: 5 μm.

Next, we analyzed the MIC60 immunosignal intensity of single synaptic mitochondria in the OPL (Figure 2). As mentioned, rod photoreceptor synapses contain only a single but large mitochondrion close to the synaptic ribbon. This presynaptic mitochondrion is well identifiable and accessible for imaging analyses. Immunolabelling with antibodies against RIBEYE served again as a reference to identify this single presynaptic mitochondrion in close vicinity to the synaptic ribbon. In agreement with single-mitochondrion resolution, we observed single round ring-like immunolabelled structures (mitochondria) in close vicinity to the synaptic ribbon, as expected (Figure 2). These ring-like structures immunolabelled with the MIC60 antibody correspond to the immunolabelled inner mitochondrial membrane. The MIC60 signal intensity of the individual synaptic mitochondria in the OPL was considerably stronger in the retinas of CFA-injected control mice in comparison to MOG/CFA-injected EAE mice (Figure 2D1,D2). The number of RIBEYE puncta in the OPL was not significantly different between MOG/CFA- and CFA-injected samples, like in a previously published study [27], indicating that the number of photoreceptor synaptic ribbons is not different between the two samples (i.e., MOG/CFA vs. CFA). In contrast, the number of MIC60 puncta in the OPL that were detectable by immunofluorescence microscopy was significantly less in the OPL of MOG/CFA-injected mice in comparison to CFA-injected control mice at day 9 post immunization (Figure 2C3,C4).

**Figure 3:**
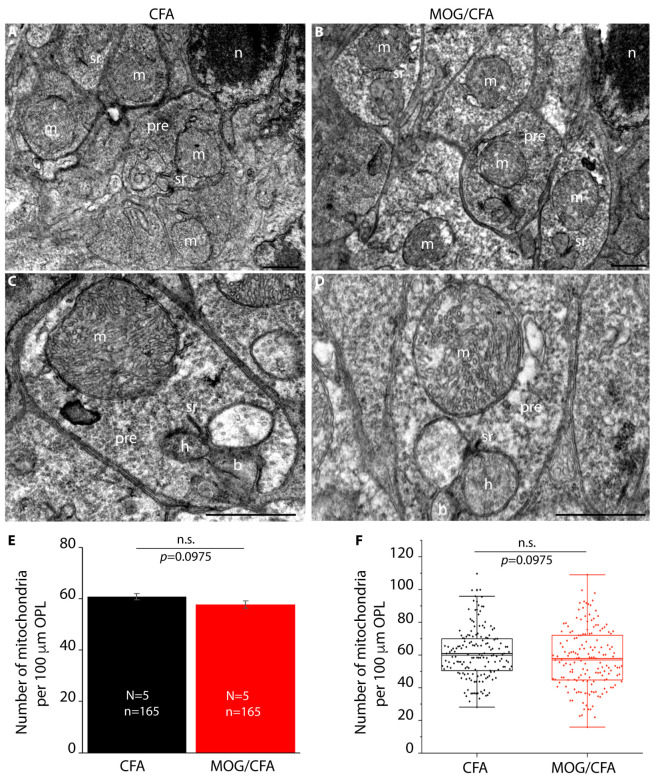
The number of mitochondria in rod photoreceptor synapses is unchanged in MOG/CFA-injected EAE mice in comparison to CFA-injected control mice at day 9 post -immunization as evaluated by transmission electron microscopy (TEM). (**A**,**B**) Representative low-magnification TEM images of rod photoreceptor synapses from CFA-injected control mice (**A**) and MOG/CFA-injected EAE mice (**B**). (**C**,**D**) Representative images of single rod photoreceptor ribbon synapse from CFA-injected control mice (**C**) and MOG/CFA-injected EAE mice (**D**) at day 9 post- immunization. The presynaptic mitochondrion (m) in close vicinity to the synaptic ribbon (sr) is clearly visible. (**E**,**F**) Quantification of the number of presynaptic mitochondria in rod photoreceptor synapses normalized to an OPL length of 100 µm. The column plots in (**E**) show normalized mean values ± S.E.M. In (**F**), the individual data points from (**E**) are given as box-and-whisker plots. Boxes represent 25th-75th percentiles; mean and median values are indicated by thick and thin horizontal lines, respectively. Whiskers are equal to 1.5 times the interquartile range (IQR). To test whether the number of mitochondria in rod photoreceptor synapses differed significantly between CFA- and MOG/CFA-injected animals, the Mann–Whitney U-test was conducted. The number of mitochondria did not differ significantly between both groups (effect size d = 0.1811; power = 0.3316). Data were considered statistically different with *p* < 0.05. Abbreviations: m, mitochondrion; sr, synaptic ribbon; pre, presynaptic; h, b, dendritic tips of horizontal and bipolar cells; CFA, complete Freund’s adjuvant; EAE, experimental autoimmune encephalomyelitis; MOG, myelin oligodendrocyte glycoprotein; TEM, transmission electron microscopy; N = number of independent experiments; n = number of TEM images analyzed. ONL, outer nuclear layer; OPL, outer plexiform layer; S.E.M., standard error of the mean; n.s., non-significant. Scale bars: 1 μm.

These latter findings suggest that either the number of mitochondria in the photoreceptor synapses of the OPL was decreased or that the MIC60 content of the individual mitochondria in the OPL was decreased in the retinas of MOG/CFA-injected mice in comparison to CFA-injected control mice. To discriminate between these possibilities, we performed transmission electron microscopy to determine the number of mitochondria in the rod photoreceptor terminals of the OPL at the ultrastructural level (Figure 3).

The electron microscopic analyses demonstrated that the number of presynaptic mitochondria in rod photoreceptor synapses of the OPL per 100 µm of OPL is not significantly different between MOG/CFA-injected EAE mice and CFA-injected control mice (Figure 3). Thus, we conclude that a strongly decreased MIC60 content of individual synaptic mitochondria is the reason for the decreased number of MIC60 puncta in the OPL, as quantified in Figure 2C3,C4.

**Figure 4:**
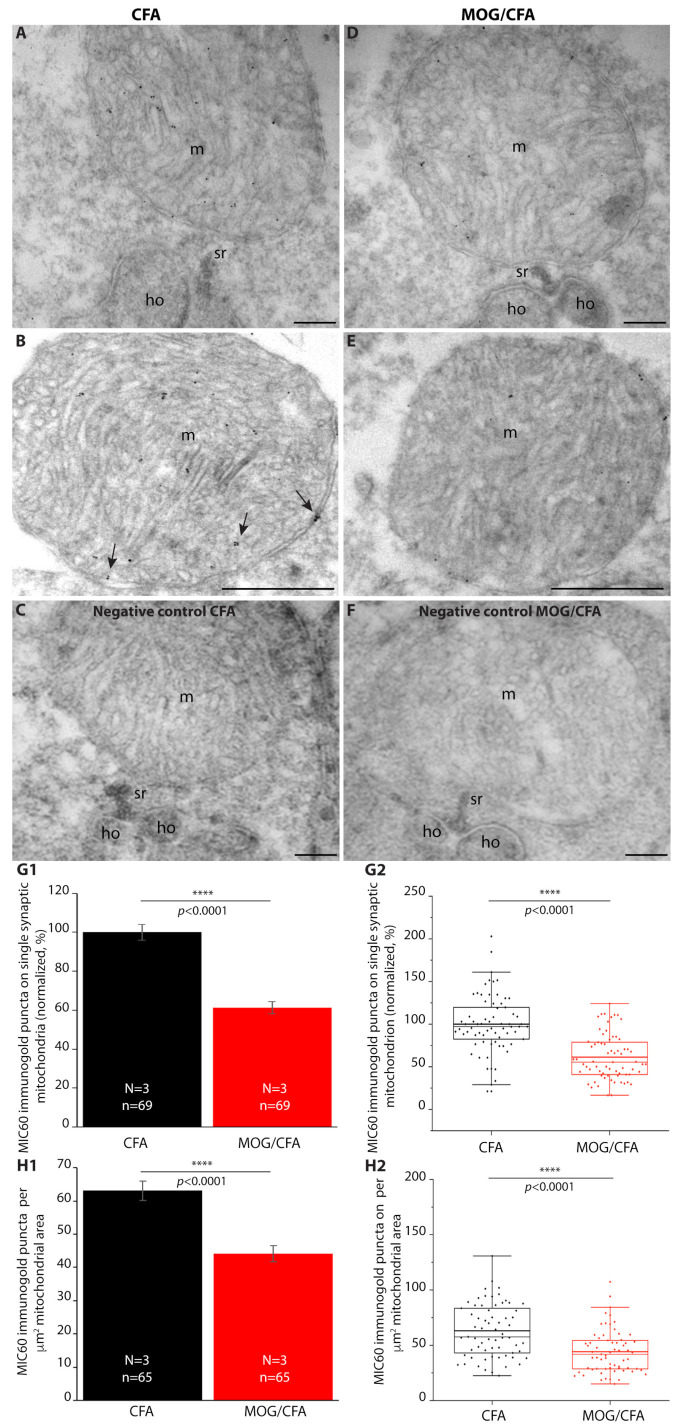
Decreased number of MIC60 immunogold puncta on presynaptic mitochondria of photoreceptor synapses from MOG/CFA-injected EAE mice in comparison to CFA-injected control mice on day 9 after immunization. Ultrathin LR Gold sections of retinas from CFA-injected control mice and MOG/CFA-injected EAE mice immunolabelled with MIC60 antibody by post-embedding immunogold electron microscopy. Presynaptic mitochondria in close vicinity to the synaptic ribbon were analyzed. (**A**,**B**) Representative TEM images of photoreceptor synapses from CFA-injected control mice immunolabelled with MIC60 antibody. (**D**,**E**) Representative images of photoreceptor synapses from MOG/CFA-injected mice immunolabelled with MIC60 antibody. (**C**,**F**) Representative negative control incubations. (**G1**,**G2**,**H1**,**H2**) Quantification of MIC60 immunogold puncta on presynaptic mitochondria of photoreceptor synapses. (**G1**) Column plots show normalized arithmetic means of immunogold puncta on presynaptic mitochondria of rod synapses ± S.E.M. The arithmetic mean of CFA control mice was set to 100%, and MOG/CFA values were compared to this reference. In (**G2**), the individual data points from (**G1**) are given as box-and-whisker plots. MIC60 immunogold puncta differed significantly between CFA- and MOG/CFA- injected animals (effect size d = 1.2789; power = 1.0). (**H1**) Column plots show normalized arithmetic mean values of MIC60 immunogold puncta per μm^2^ presynaptic mitochondria of presynaptic rod terminals ± S.E.M. In (**H2**), the individual data points from (**H1**) are given as box-and-whisker plots. In the box-and-whiskers plots in (**G2**,**H2**), boxes represent 25th–75th percentiles; mean and median values are indicated by thick and thin horizontal lines, respectively. Whiskers are equal to 1.5 times the interquartile range (IQR). A comparison of MIC60 signals for individual mitochondria on ultrastructural level was performed with a Mann–Whitney U-test. MIC60 immunogold puncta differed significantly between CFA- and MOG/CFA -injected animals (effect size d = 0.8618; power = 0.9949). Data were considered statistically different with *p* < 0.05. Abbreviations: CFA, complete Freund’s adjuvant; EAE, experimental autoimmune encephalomyelitis; MOG, myelin oligodendrocyte glycoprotein; m, mitochondrion; sr, synaptic ribbon; ho, dendritic tip of horizontal cell; TEM, transmission electron microscope; N = number of independent experiments; n = number of mitochondria; ****, *p* < 0.0001. Arrows point to individual immunogold puncta. Scale bars: 200 nm (**A**,**C**,**D**,**F**); 500 nm (**B**,**E**).

This conclusion is further supported by post-embedding immunogold analyses with the MIC60 antibody at the electron microscopical level (Figure 4). Post-embedding immunogold electron microscopy revealed that the MIC60 immunogold labelling density of presynaptic mitochondria of rod photoreceptor synapses is higher in CFA-injected control mice than in MOG/CFA-injected EAE mice. Immunogold electron microscopy provides clear evidence that the presynaptic mitochondria in close proximity to the synaptic ribbon in rod photoreceptor synapses were affected by EAE. The presynaptic mitochondria contained significantly less MIC60 immunogold particles in MOG/CFA-injected animals than in control-injected animals (Figure 4G1,G2,H1,H2). The MIC60 immunogold data confirm the MIC60 immunofluorescence data (Figure 1 and Figure 2).

**Figure 5:**
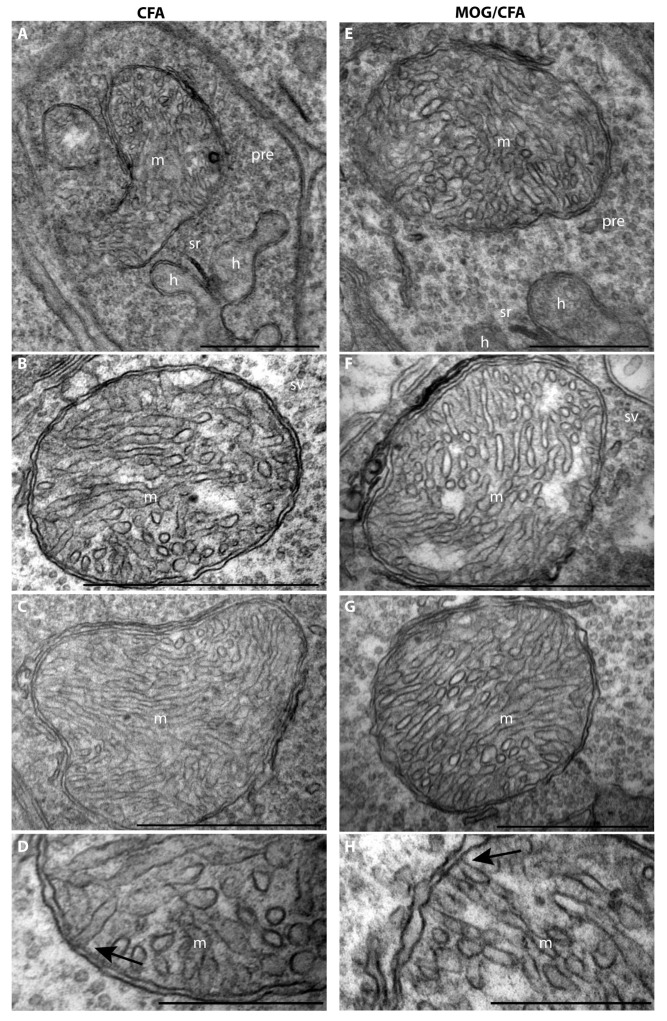
The ultrastructure of presynaptic mitochondria in rod photoreceptor synapses of EAE retinas is unchanged in comparison to control synaptic mitochondria at day 9 post-immunization. (**A**,**E**) Lower-magnification TEM images of presynaptic mitochondria close to the synaptic ribbon in rod photoreceptor ribbon synapses of CFA-injected mice (**A**) and MOG/CFA-injected EAE mice (**E**), respectively. Further examples of presynaptic mitochondria of photoreceptor synapses from CFA-injected control mice (**B**–**D**) and MOG/CFA-injected EAE mice (**F**–**H**) at high magnification. No obvious ultrastructural alterations of the presynaptic mitochondria were observed. Normal appearing mitochondrial contact sites are present in the synaptic mitochondria of photoreceptor synapses from MOG/CFA-injected EAE mice (arrow in **H**) and in mitochondria from CFA-injected control mice (arrow in **D**). Abbreviations: CFA, complete Freund’s adjuvant; EAE, experimental autoimmune encephalomyelitis; MOG, myelin oligodendrocyte glycoprotein; m, mitochondrion; sr, synaptic ribbon; h, horizontal cell dendrite; pre, presynaptic. Scale bars: 1 μm (**A**–**C**;**E**–**G**); 500 nm (**D**,**H**).

Since MIC60 is important for the MICOS contact sites in mitochondria [95,103], we analyzed the mitochondria at higher magnifications to identify possible mitochondrial cristae defects, as they were observed in MIC60-deficient yeasts and drosophila [103,104]. Surprisingly, we did not observe gross mitochondrial ultrastructural abnormalities (Figure 5). Also, the contact sites between the inner and outer mitochondrial membrane appeared normal and unaltered (Figure 5). From this, we conclude that the remaining amount of MIC60 in the synaptic mitochondria of MOG/CFA-injected mice is still sufficient to preserve the mitochondrial contact sites. Alternatively, MIC60 might be less important for the MICOS contact sites and cristae architecture in photoreceptor presynaptic mitochondria and/or functionally replaced with another protein.

**Figure 6:**
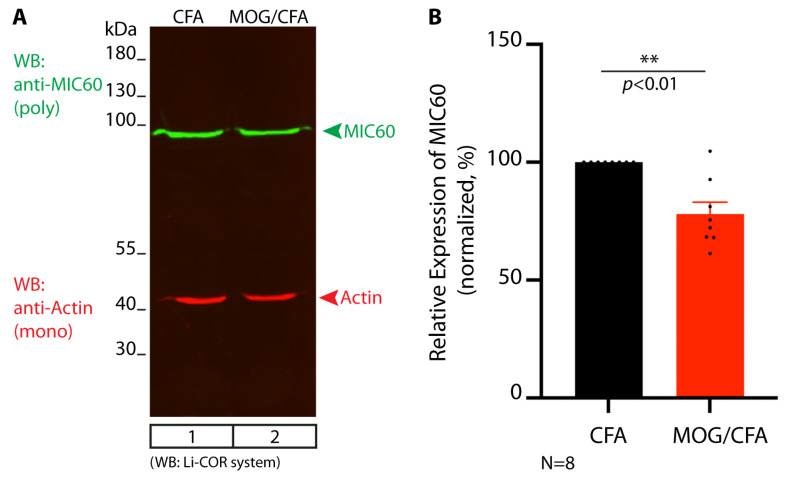
Total expression of MIC60 protein in retinal lysates from MOG/CFA-injected EAE mice is significantly diminished in comparison to retinal lysates from CFA-injected control mice. Pairs of retinas from 8 independent injections were isolated from MOG/CFA-injected EAE mice and CFA-injected control mice at day 9 post-immunization. Isolated retinas were dissolved in Laemmli SDS sample buffer and probed by Western blot (WB) with antibodies against MIC60 and actin. (**A**) show exemplary representative WB examples. WBs were analyzed with the LI-COR Odyssey system. MIC60 band intensities (**bands in green**) were normalized to the corresponding actin band intensities (**bands in red**), which served as a loading control. Normalized MIC60 values from the 8 different experiments (pairs of retinas from CFA- and MOG/CFA-injected mice) were quantified in (**B**). Normalized CFA values were set to 100% for better assessment of the relative differences between CFA-injected control mice and MOG/CFA-injected EAE mice. The column plots show arithmetic means ± SEMs. A significant reduction in global MIC60 expression was observed in the MOG/CFA-injected retinas. One-sample Student’s *t*-test was used for statistical analysis (effect size d = 1.5271; power = 0.9999 for MIC60 WBs; effect size d = 0.0482; power = 0.0721 for actin WBs). *p* < 0.05 was considered statistically significant. Abbreviations: CFA, complete Freund’s adjuvant; MOG, myelin oligodendrocyte glycoprotein; EAE, experimental autoimmune encephalomyelitis; SDS, sodium dodecylsulfate; N = number of independent experiments; S.E.M., standard error of the mean; **, *p* < 0.01.

We also tested global MIC60 expression in the retina by Western blot (WB) analyses and found a significant decrease in MIC60 expression in retinal lysates from MOG/CFA-injected retinas in comparison to retinal lysates from CFA-injected mice at day 9 post- immunization (Figure 6).

**Figure 7:**
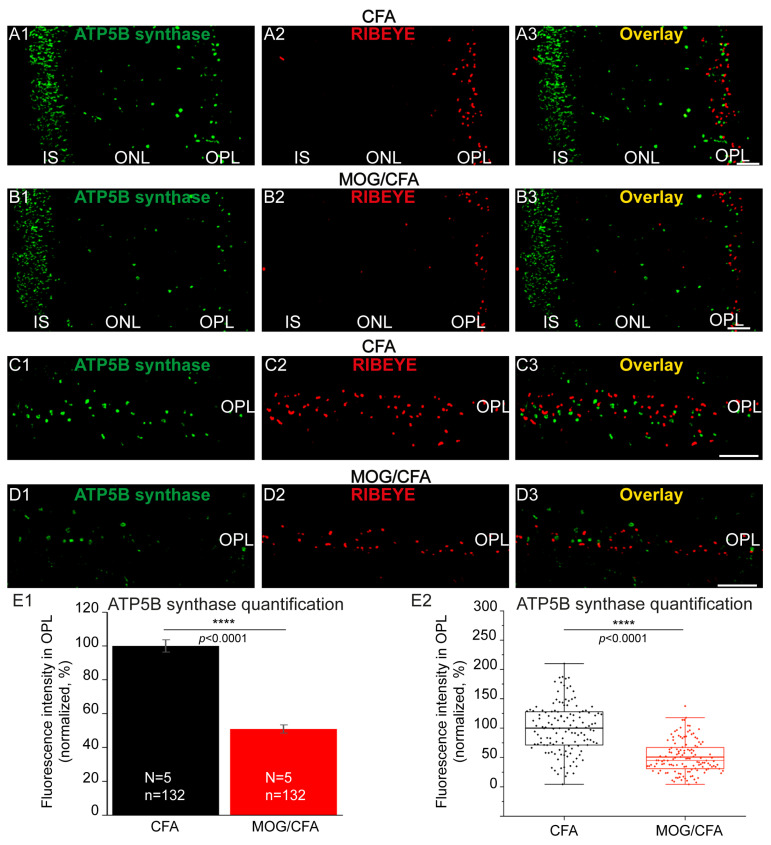
Decreased expression of the mitochondrial ATP5B synthase subunit of the F_1_F_0_ ATP synthase complex in synaptic mitochondria of photoreceptor synapses in MOG/CFA-injected EAE retinas at day 9 post-immunization. The 0.5 µm thin retina sections from CFA-injected mice and MOG/CFA-injected mice were incubated with mouse monoclonal ATP5B antibody (**A1**,**B1**,**C1**,**D1**) and with rabbit polyclonal monoclonal antibodies against RIBEYE (U2656) (**A2**,**B2**,**C2**,**D2**), as indicated. Overlay images are shown in (**A3**,**B3**,**C3**,**D3**). (**A1**–**A3**,**B1**–**B3**) show representative low-magnification micrographs of the photoreceptor compartments from MOG/CFA- and CFA-injected mice. The immunolabelled OPL is magnified in (**C1**–**C3**,**D1**–**D3**). (**E1**,**E2**) ATP5B immunosignals from the OPL were quantified as integrated density from 5 different littermate mice at day 9 post-injection. (**E1**) shows column plots in which the arithmetic mean values ± S.E.M.s are depicted. MOG/CFA integrated density immunofluorescence values were normalized to the mean of the CFA control values. In (**E2**), the same data as shown in (**E1**) are depicted as box-and-whisker plot to show the individual datapoints. In (**E2**), boxes represent 25th–75th percentile of the data points, with the mean and median values indicated by thick and thin horizontal lines, respectively, and whiskers are equal to 1.5 times the interquartile range (IQR). Mitochondrial ATP synthase 5B subunit expression is significantly reduced in the experimental group in comparison to the CFA control group as revealed by the Mann–Whitney U-test (effect size d = 1.3938; power = 1.0). *p*-values < 0.05 were considered statistically significant. Abbreviations: ATP5B, mitochondrial ATP synthase subunit 5B of the mitochondrial F_1_F_0_ ATP synthase complex; CFA, complete Freund’s Adjuvant; EAE, experimental autoimmune encephalomyelitis IS, inner segment; MOG, myelin oligodendrocyte glycoprotein; ONL, outer nuclear layer; OPL, outer plexiform layer; S.E.M., standard error of the mean; N = number of independent experiments; n = number of images analyzed from retinal sections; ****, *p* < 0.0001. Scale bars: 5 μm.

Several other mitochondrial proteins showed a similar change in mitochondrial signal strength in immunofluorescence microscopy as described above for MIC60. These proteins that showed a decreased immunofluorescence signal in synaptic mitochondria of photoreceptors from MOG/CFA-injected EAE animals in comparison to CFA-injected control animals included the ATP-synthase 5B subunit, a component of the mitochondrial F_1_F_0_-ATPase complex, cytochrome c oxidase subunit 1 (COX1), and Pink1. As depicted in Figure 7, the ATP-synthase 5B subunit showed a decreased expression in photoreceptor synapses in the OPL of retinas from MOG/CFA-injected mice in comparison to photoreceptor synapses from CFA-injected control mice (Figure 7).

**Figure 8:**
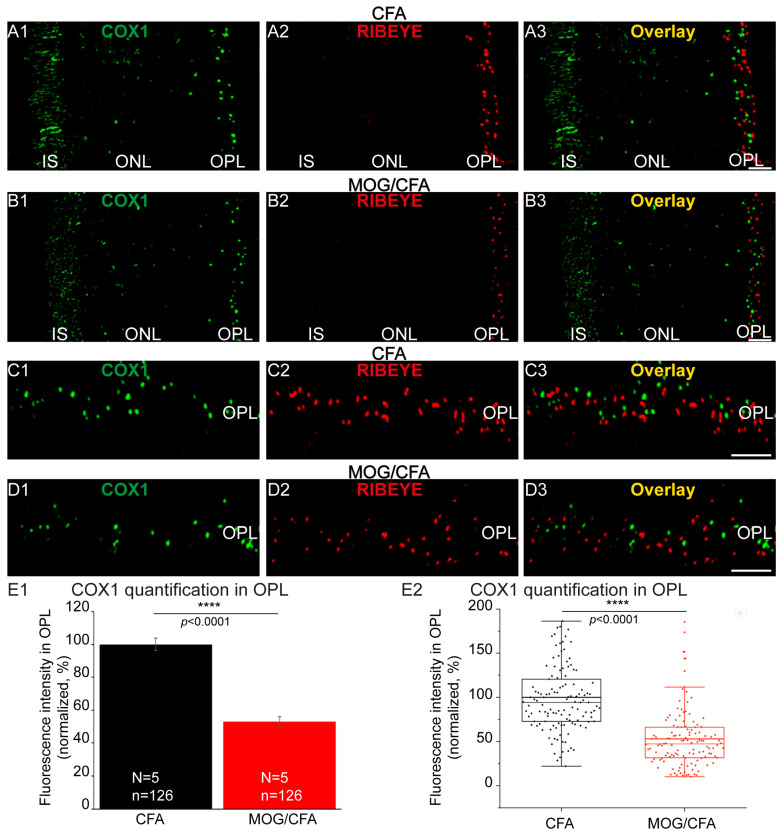
Decreased expression of the mitochondrial COX1 protein in synaptic mitochondria of photoreceptor synapses in MOG/CFA-injected EAE retinas at day 9 post-immunization. The 0.5 µm thin retina sections from CFA-injected mice and MOG/CFA-injected mice were incubated with rabbit polyclonal antibody against COX1 (**A1**,**B1**,**C1**,**D1**) and with mouse monoclonal antibody against RIBEYE (2D9) (**A2**,**B2**,**C2**,**D2**), as indicated. Overlay images are shown in (**A3**,**B3**,**C3**,**D3**). (**A1**–**A3**,**B1**–**B3**) show low-magnification micrographs of the photoreceptor compartments. The immunolabelled OPL is magnified in (**C1**–**C3**,**D1**–**D3**). (**E1**,**E2**) COX1 immunosignals from the OPL were quantified as integrated density from 5 different littermate mice at day 9 post-injection. (**E1**) shows column plots of the arithmetic mean values ± S.E.M.s. MOG/CFA integrated density immunofluorescence values were normalized to the mean of the CFA control values that was set to 100%. (**E2**) Same data as in (**E1**) depicted as box-and-whisker plot to show the individual datapoints and their distribution. (**E2**) Boxes represent 25th–75th percentile of the data points, and mean and median values are indicated by thick and thin horizontal lines, respectively. Whiskers are equal to 1.5 times the interquartile range (IQR). Comparison of COX1 expression between control and experimental groups using a Mann–Whitney U-test revealed significant reduction in COX1 expression in the OPL of MOG/CFA-injected animals (effect size d = 1.2242; power = 1.0). *p*-values < 0.05 were considered statistically significant. Abbreviations: CFA, complete Freund’s Adjuvant; COX1, cytochrome c oxidase subunit 1; EAE, experimental autoimmune encephalomyelitis IS, inner segment; MOG, myelin oligodendrocyte glycoprotein; ONL, outer nuclear layer; OPL, outer plexiform layer; S.E.M., standard error of the mean; N = number of independent experiments; n = number of images analyzed from retinal sections; ****, *p* < 0.0001. Scale bars: 5 μm.

Cytochrome c oxidase subunit 1 (COX1) is the main component of the cytochrome-c-oxidase complex (complex IV) and of central importance for mitochondrial electron transport chain and oxidative phosphorylation [105,106]. We found that COX1 expression was impaired in synaptic mitochondria of photoreceptor synapses in the OPL of MOG/CFA-injected EAE mice on day 9 post-immunization in comparison to photoreceptor synapses of CFA-injected mice (Figure 8).

**Figure 9:**
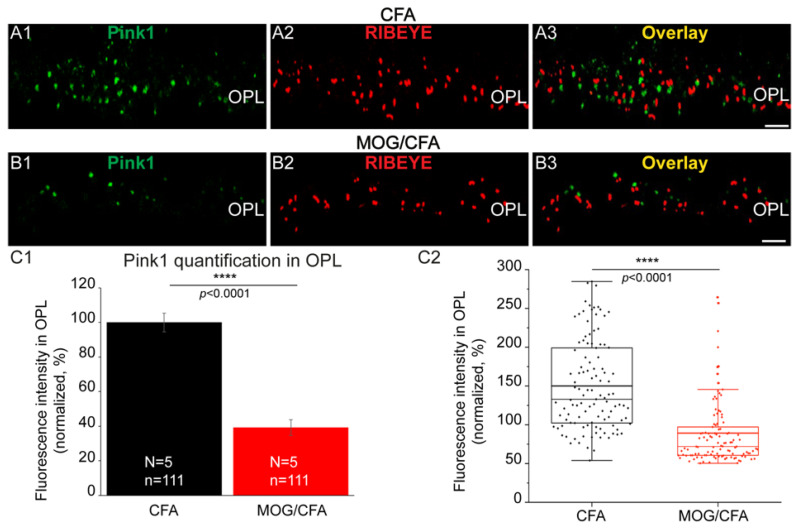
Strong decrease in Pink1 immunosignals in the OPL of MOG/CFA-injected mice in comparison to CFA-injected control mice on day 9 post-injection. The 0.5 µm-thin retina sections from both groups (i.e., CFA (**A1**–**A3**) and MOG/CFA (**B1**–**B3**)) were double-immunolabeled with rabbit polyclonal antibody against Pink1 (**A1**,**B1**) and mouse monoclonal antibody against RIBEYE (2D9) (**A2**,**B2**). Signals from the green (**A1**,**B1**) and red (**A2**,**B2**) channels were merged in (**A3**,**B3**). The double-immunolabelling data demonstrate that Pink1 puncta are located close to the RIBEYE puncta (**A3**,**B3**). (**C1**,**C2**) Quantification of the Pink1 immunosignals in the OPL. Column plots in (**C1**) show normalized arithmetic mean fluorescence values (integrated densities) ± S.E.M. Pink1 immunosignals in the OPL of MOG/CFA-injected mice were normalized to the arithmetic mean of the values from CFA-injected control mice (that was set to 100%). The Mann–Whitney U-test was used to determine whether Pink1 is significantly altered in MOG/CFA-injected animals in comparison to CFA-injected control animals because data were non-normally distributed (effect size d = 1.1445; power = 1.0). *p*-values < 0.05 were considered statistically significant. The same data as shown in (**C1**) are also depicted as box-and-whisker plots in (**C2**) to show the individual datapoints. Boxes represent the 25th–75th percentile of the data points. The mean and median values in the box-and-whisker plot are indicated by thick and thin horizontal bars, respectively. Whiskers are equal to 1.5 times the interquartile range (IQR). Abbreviations: CFA, complete Freund’s adjuvant; Pink1, PTEN-induced kinase 1; MOG, myelin oligodendrocyte glycoprotein; OPL, outer plexiform layer; S.E.M., standard error of the mean; N = number of independent experiments; n = number of images analyzed from retinal sections; ****, *p* < 0.0001. Scale bars: 5 μm.

The PTEN-induced kinase 1 (Pink1) is a mitochondria-associated kinase linked to familial Parkinson’s disease [107]. Pink1 is a putative neuroprotective protein preventing mitochondrial dysfunction [108,109] but also serves as a mitochondrial stress sensor protein that is involved in mediating the autophagy of damaged mitochondria [110,111,112]. Using qualitative and quantitative immunofluorescence microscopy, we observed that Pink1 expression on synaptic mitochondria of photoreceptor synapses from MOG/CFA-injected mice was decreased in comparison to that in photoreceptor synapses of CFA-injected control mice on day 9 after immunization (Figure 9).

**Figure 10:**
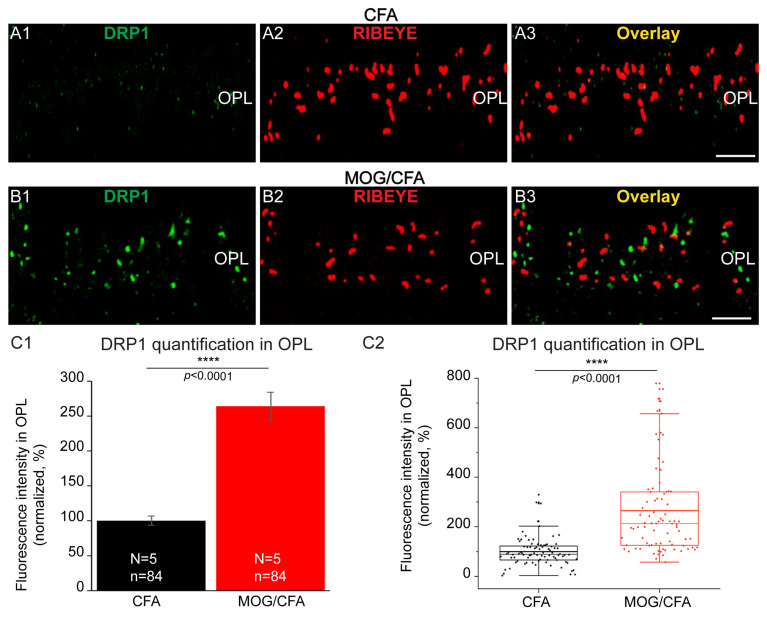
DRP-1 immunosignals in synaptic mitochondria of photoreceptor synapses are strongly increased in MOG/CFA-injected EAE mice compared to CFA-injected control mice on day 9 after immunization. The 0.5 µm-thin retina sections from CFA mice (**A1**–**A3**) and MOG/CFA mice (**B1**–**B3**) were double-immunolabeled with rabbit polyclonal antibody against DRP1 (**A1**,**B1**) and mouse monoclonal antibody against RIBEYE (2D9) (**A2**,**B2**). Signals from the green (**A1**,**B1**) and red (**A2**,**B2**) channels were merged in (**A3**,**B3**). Quantification of DRP1 immunosignals in the OPL is depicted in (**C1**,**C2**). Column plots in (**C1**) show normalized arithmetic mean fluorescence values (integrated densities) ± S.E.M. DRP1 immunosignals in the OPL of MOG/CFA-injected mice were normalized to the arithmetic mean of the values from CFA-injected control mice (that was set to 100%). Statistical analysis using the Mann–Whitney U-test of the non-normally distributed data revealed a significant increase in DRP1 immunosignals in the OPL of MOG/CFA-injected animals in comparison to in the CFA control group (effect size d = 1.2141; power = 0.9999). *p*-values < 0.05 were considered statistically significant. The same data as shown in (**C1**) are depicted in (**C2**) as box-and-whisker plots to show the individual datapoints. Boxes represent the 25th–75th percentile of the data points. The mean and median values in the box-and-whisker plot are indicated by thick and thin horizontal bars, respectively. Whiskers are equal to 1.5 times the interquartile range (IQR). Abbreviations: CFA, complete Freund’s adjuvant; DRP1, dynamin-related protein 1; EAE, experimental autoimmune encephalomyelitis; MOG, myelin oligodendrocyte glycoprotein; OPL, outer plexiform layer; S.E.M., standard error of the mean; ****, *p* < 0.0001; N = number of independent experiments; n = number of images analyzed from retinal sections. Scale bars: 5 μm.

Thus, many functionally relevant mitochondrial proteins were less abundant in synaptic mitochondria of retinas from MOG/CFA-injected mice. But not all mitochondria-associated proteins were less strong in signal strength in synaptic mitochondria of EAE mice on day 9 after injection. In contrast, we found that dynamin-related protein 1 (DRP1), as an important mitochondria-associated protein, was increased at synaptic mitochondria of photoreceptor synapses in EAE (Figure 10). DRP1 is involved in mitochondrial fragmentation in many disease-related conditions [110,113,114,115,116,117,118]. Mitochondrial fission and fragmentation are often considered consequences of mitochondrial dysfunction [114,118]. We found increased DRP1 expression in photoreceptor synaptic mitochondria in the OPL of MOG/CFA-injected EAE mice in comparison to those from CFA-injected control mice as evaluated by immunofluorescence microscopy (Figure 10).

**Figure 11:**
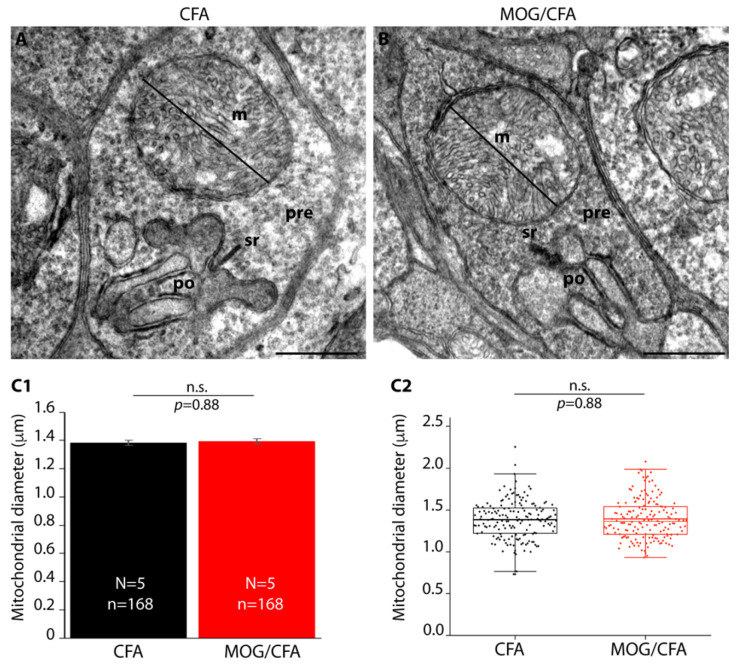
The diameter of synaptic mitochondria in photoreceptor synapses is not yet altered at day 9 after immunization in MOG/CFA-injected EAE mice in comparison to CFA-injected control mice. Transmission electron micrographs from photoreceptor mitochondria of CFA-injected mice (representative rod photoreceptor synapse shown in (**A**)) and MOG/CFA-mice (representative photoreceptor synapse shown in (**B**)) were acquired at a primary magnification of 8.200X. The diameter was measured in a blinded manner with ImageJ as schematically depicted via the black lines in (**A**,**B**). The scale bar provided by the electron microscope software suite served as a length reference. The diameter of the mitochondria is depicted in (**C1**,**C2**). In the column plots in (**C1**), arithmetic means ± S.E.M. are depicted. The Mann–Whitney U-test was conducted from the non-normally distributed data to determine if there were significant differences in the diameter of synaptic mitochondria between the CFA and MOG/CFA groups. The diameter of mitochondria did not significantly differ between both groups (effect size d = 0.0425; power = 0.065). *p*-values < 0.05 were considered statistically significant. In (**C2**), the values from C1 are shown as box-and-whisker plot to document the individual values. Boxes represent the 25th-75th percentile of the data points. The mean and median values in the box-and-whisker plot are indicated by thick and thin horizontal bars, respectively. Whiskers are equal to 1.5 times of the interquartile range (IQR). Abbreviations: CFA, complete Freund’s adjuvant; EAE, experimental autoimmune encephalomyelitis; MOG, myelin oligodendrocyte glycoprotein; m, mitochondrion; pre, presynaptic; po, postsynaptic; sr, synaptic ribbon; N = number of independent experiments; n = number of mitochondria; n.s., non-significant. Scale bars: 500 nm (**A**,**B**).

Since DRP1 is involved in mitochondrial fission, we semi-quantitatively evaluated the diameter of synaptic mitochondria by transmission electron microscopy (Figure 11).

We interpret this finding that the increased association of DRP1 with EAE synaptic mitochondria at this early time point has not yet led to a fragmentation of mitochondria. The fragmentation of mitochondria is a time-consuming, multi-step process [115,119,120,121,122], and this process might not have yet been completed in the photoreceptor synapse at the presynaptic mitochondrion at that early time point of pre-clinical EAE (see Discussion).

**Figure 12:**
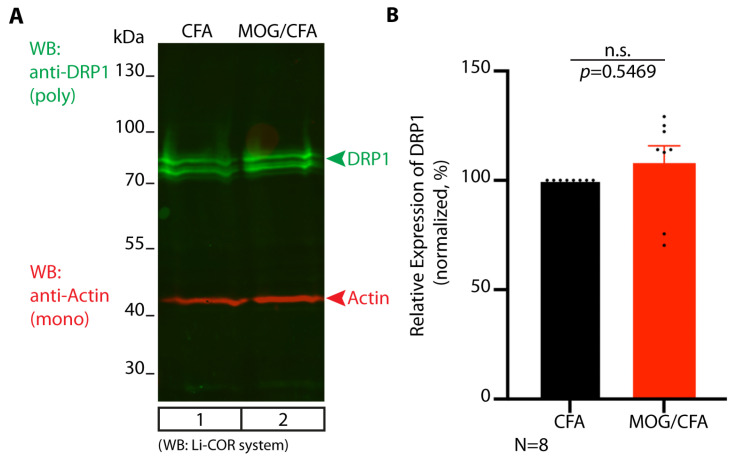
No changes in global expression of DRP-1 protein in retinal lysates of MOG/CFA-injected EAE mice in comparison to CFA-injected control mice at day 9 after immunization, as evaluated by Western blot analyses. Retinas isolated from both groups of mice (CFA-injected vs. MOG/CFA-injected) on day 9 after immunization were directly lysed in SDS/Laemmli buffer and probed for the expression of DRP1 by Western blot analyses (green channel). Afterward, membranes were re-probed with Actin antibody (red channel). WB immunosignals were recorded with a LI-COR Odyssey system using fluorescent secondary antibodies. In (**A**), green and red channels were overlayered electronically. The DRP1 band intensities were normalized to actin that served as a loading control. In each set of experiments that contained a sample from a MOG/CFA-injected and a CFA-injected animal, the CFA values were set to 100% for better comparability. Quantified DRP1 band intensities are displayed in (**B**). Column plots in (**B**) show normalized arithmetic mean fluorescence values (integrated densities) ± S.E.M. The one-sample Wilcoxon-test was used for statistical analysis (effect size d = 0.3524; power = 0.3432 for DRP1 WBs; effect size d = 0.0482; power = 0.0721 for Actin WBs). *p*-values < 0.05 were considered statistically significant. We did not observe a statistically significant difference in the global expression of DRP1 between MOG/CFA-injected samples vs. CFA-injected samples by the WB analyses. Abbreviations: CFA, complete Freund’s adjuvant; MOG, myelin oligodendrocyte glycoprotein; EAE, experimental autoimmune encephalomyelitis; DRP1, dynamin-related protein 1; S.E.M., standard error of the mean; N = number of independent experiments; n.s., non-significant; SDS, sodium dodecylsulfate; WB, Western blot.

We also analyzed the global expression of DRP1 in the retina with Western blot analyses of total retinal lysates obtained from retinas of MOG/CFA-injected mice and CFA- injected mice at day 9 after immunization. We found that the global expression of DRP1 was not altered and did not differ between the retinas of CFA-injected mice in comparison to MOG/CFA-injected mice on day 9 after immunization (Figure 12).

We interpret the lack of global changes in DRP1 expression in early EAE retinas based on our observation that synaptic mitochondria are more strongly affected at early pre-clinical stages of EAE than the majority of retinal mitochondria, e.g., mitochondria in the inner segments of photoreceptors (see the Discussion).

## 4. Discussion

Mitochondria play a central role in energy metabolism of the nervous system, apart from additional important roles in metabolic and signalling pathways [123,124,125,126,127]. Adequate energy production is particularly relevant for the central nervous system (CNS), which includes the retina. At rest, the CNS consumes ~20% of oxygen but represents only ~2% of human body weight [41,128]. In neurons, synapses and synaptic activity require high levels of energy equivalents (adenosine triphosphate; ATP) and thus particularly depend upon proper mitochondrial function [33,34,35,129,130]). Synaptic activity requires local ATP synthesis that is largely provided by the Ca^2+^-dependent regulation of mitochondria- and glycolysis-mediated ATP production [33,129,131]. Previous studies showed that mitochondrial ATP production is modulated by activity in a Ca^2+^-dependent manner [130,132]. Appropriate ATP production is important also for the retina and for the continuously active photoreceptor ribbon synapses [49,133,134,135,136,137,138].

Previous observations already suggested an important role of mitochondria in the pathogenesis of multiple sclerosis, particularly at the advanced clinical/chronic stages of the disease [41,139,140]. Similarly, mitochondrial dysfunctions have also been found in the EAE mouse model of MS, predominantly at advanced stages [47,48,140,141].

In our previous studies on the retina, we observed morphological and functional alterations in retinal synapses, particularly in photoreceptor synapses, in the EAE mouse model of MS [25,26,27,29,53]. These changes occurred at an early stage (i.e., on day 9 after injection), at which no obvious alterations of the myelinated axons of the optic nerve and no signs of optic neuritis were observed [25].

In the present study, we analyzed whether these early synapse dysfunctions correlate with alterations of synaptic mitochondria. Additionally, we compared EAE/MS-induced effects on mitochondrial protein composition between the presynaptic mitochondria of photoreceptor synapses with non-synaptic mitochondria (i.e., mitochondria in the inner segments of photoreceptors).

First, we identified changes in the mitochondrial protein composition in presynaptic mitochondria of rod photoreceptor synapse from EAE mice in comparison to CFA-injected control mice. These changes already occurred at a remarkably early stage of pre-clinical EAE. We found that the expression of many functionally relevant mitochondrial proteins (e.g., MIC60, COX1, Pink1, and the ATP-synthase 5B subunit) were decreased in photoreceptor synaptic mitochondria already on day 9 after injection. The decreased expression of these functionally relevant proteins indicates a functional impairment of synaptic mitochondria in photoreceptor synapses of EAE mice and most likely decreased functional activity. Clearly, a functional impairment remains to be investigated by future investigations. Our data of decreased mitochondrial protein expression in synaptic photoreceptor mitochondria are in line with previously published data that demonstrated decreased levels of mitochondrial gene transcripts in the spinal cord of EAE mice [47,48].

Of note, not all the analyzed mitochondrial proteins were less strongly expressed in photoreceptor synaptic mitochondria of EAE mice on day 9 after injection. In contrast, the mitochondria-associated protein DRP1 was strongly enriched at photoreceptor synaptic mitochondria of MOG/CFA-injected EAE mice in comparison to CFA-injected control mice. DRP1 is involved in the fission of mitochondria [115,119,120,121,122,142,143]. The fission of mitochondria is often considered a consequence of mitochondrial dysfunction and has been frequently observed in neurodegenerative diseases [142,144,145]. Our electron microscopic analyses on the diameter of the presynaptic mitochondrion did not show a difference between MOG/CFA-injected EAE mice and CFA-injected control mice. From this finding, we conclude that the increased association of DRP1 with EAE synaptic mitochondria has not yet led to a fragmentation of mitochondria at this early time point on day 9 after immunization in the pre-clinical phase of EAE. The fragmentation of mitochondria is a time-consuming, multi-step process [115,119,120,121,122,146,147], and this process might not have yet been completed in the photoreceptor synapse 9 days after immunization.

Second, we demonstrate that mitochondria in photoreceptors are not equally affected by EAE but are differentially impaired even within the same cell. In photoreceptors, the bulk of mitochondria are located within the inner segments at a large distance from the presynaptic terminal [49]. As discussed above, presynaptic mitochondria in rod photoreceptor synapses showed a decreased expression of MIC60. In contrast, MIC60 expression in mitochondria in photoreceptor inner segments were unaltered although these mitochondria are contained in the same cell. Thus, the presynaptic mitochondria appear to be more sensitive to EAE than inner segment mitochondria as judged by the differential effect of EAE on MIC60 expression in synaptic and extra-synaptic mitochondria. Our data are in line with previous data from other systems that demonstrated molecular and functional heterogeneity of mitochondria between different organs, tissues, and individual cells [129,148,149,150,151] and even within the same cell [152,153,154,155,156,157].

What could be the reason for the differential sensitivity of photoreceptor mitochondria, i.e., the increased vulnerability of the presynaptic mitochondria in comparison to mitochondria in photoreceptor inner segments? Physiologically, activity-dependent increases in presynaptic Ca^2+^ adapt presynaptic mitochondrial ATP synthesis to the increased energy demands during synaptic activity. On the other hand, a chronic overload of mitochondria with Ca^2+^ is known to damage mitochondria and leads to mitochondrial dysfunction [158,159,160,161,162]. It has been previously shown that if mitochondria are overloaded with Ca^2+^, they can render synaptic activity toxic [163]. Chronic mitochondrial Ca^2+^ overload can also lead to the opening of the mitochondrial permeability transition pore (mPTP), depolarization of mitochondrial membrane potential, mitochondrial swelling, and finally cell death [158,159,160,161].

Previous studies demonstrated increased basal presynaptic Ca^2+^-levels in photoreceptor synapses at early stages of EAE (i.e., 9 days after immunizations) at resting conditions [26]. Elevated presynaptic Ca^2+^-levels were found to be based on a decreased expression of plasma membrane Ca^2+^-ATPases (PMCA) proteins [26]. As a result, the presynaptic mitochondrion needs to cope with an increased basal presynaptic Ca^2+^ in EAE photoreceptor terminals already at rest. On top of the elevated basal presynaptic Ca^2+^-levels at rest, the activity-dependent increase in presynaptic Ca^2+^ at the continuously active photoreceptor ribbon synapse could further lead to mitochondrial damage, particularly if presynaptic Ca^2+^ extrusion mechanisms are compromised [26]. Thus, we propose that the increased Ca^2+^-load in the presynaptic photoreceptor terminals is responsible for the alterations of presynaptic mitochondria observed in the present study. Mitochondria in photoreceptor inner segments, which are located at a large distance from the presynaptic terminals, will be less affected by disturbances of presynaptic Ca^2+^-handling than mitochondria located in the presynaptic terminals. This could explain why MIC60 expression in mitochondria of photoreceptor inner segments is not altered, whereas MIC60 expression in photoreceptor synaptic mitochondria was strongly impaired. The precise molecular mechanisms remain to be elucidated by future investigations.

## 5. Conclusions and Outlook

Our data show an early impairment of functionally relevant mitochondrial proteins in presynaptic mitochondria of photoreceptor synapses in early pre-clinical EAE, which could contribute to the previously observed synaptic pathology in the retina. It will be interesting to evaluate in future studies whether the altered protein composition of synaptic mitochondria in early EAE identified in the present study will also result in different functional properties of the synaptic mitochondria. This research could help to alleviate synaptic pathology in early EAE. Early-onset mitochondrial dysfunctions in the context of neuroinflammation and neurodegeneration could link sub-cellular dysfunctions to later clinical symptoms. Interestingly, alterations of mitochondria and mitochondrial dysfunctions have also been observed in other diseases in which neurodegeneration and/or neuroinflammation play an important role, e.g., in Alzheimer’s disease, ageing, autism spectrum disorders, amyotrophic lateral sclerosis (ALS), and others [164,165,166,167,168,169]. At least in some of these diseases, the retina has already been observed to be compromised, most likely because of its high energy demand and resulting high susceptibility to mitochondrial malfunctions [170,171,172,173,174].

## Figures and Tables

**Table 1:** Primary antibodies.

Antibody	Source	References	Dilution
RIBEYE(B), rabbit polyclonal (U2656)	Lab-made	[32]	1:1000 (IF)
RIBEYE(B), mouse monoclonal (clone 2D9)	Lab-made	[25,62]	1:1000 (IF)
MIC60/Mitofilin (affinity-purified rabbit polyclonal)	Proteintech (Rosemont, IL, USA), 10179-1-AP	[63,64,65]	1:1000 (IF) 1:1000 (LI-COR) 1:100 (IG)
ATP5B (mouse monoclonal antibody, clone E1), directed against the ATP5B subunit of the F1 complex of the mitochondrial ATPase	Santa Cruz (Dallas, TX, USA) sc-55597	[66,67,68,69]	1:20 (IF)
PTEN-induced kinase-1 (PINK1), affinity-purified rabbit polyclonal antibody, 23274-1-AP	ProteinTech, 23274-1-AP	[70,71,72]	1:100 (IF)
Cytochrome c oxidase subunit 1 (COX1), aa401-aa500 affinity-purified rabbit polyclonal	Bioss Antibodies (Woburn, MA, USA) (via Biozol) (bs-3953R)	[73]	1:100 (IF)
Dynamin-related protein 1 (DRP1), raised against a C-terminal fusion protein of DRP1, affinity-purified rabbit polyclonal antibody	Proteintech 12957-1-AP	[74,75,76,77]	1:100 (IF) 1:250 (LI-COR)
Actin (mouse monoclonal antibody, clone C4), #1501R	Millipore (Burlington, MA, USA) #1501R	[78]	1:1000 (LI-COR)

IF, immunofluorescence microscopy; Western blot (WB); LI-COR, LI-COR Odyssey system, IG, immunogold.

**Table 2:** Secondary antibodies.

Antibody	Source	Dilution
Chicken anti-mouse-Alexa488	Invitrogen, Molecular Probes (Waltham, MA, USA), A-21200	1:1000 (IF)
Donkey anti-rabbit-Alexa568	Invitrogen, Molecular Probes, A-10042	1:1000 (IF)
Chicken anti-rabbit-Alexa488	Invitrogen, Molecular Probes, A-21441	1:1000 (IF)
Donkey anti-mouse-Alexa568	Invitrogen, Molecular Probes, A-10037	1:1000 (IF)
IRDye^®^ 800cw Donkey anti-rabbit	LI-COR, 926-32213	1:5000 (LI-COR)
IRDye^®^ 680 LT Donkey anti-mouse	LI-COR, 926-68022	1:5000 (LI-COR)
Goat anti-rabbit secondary antibody conjugated to 5 nm gold particles	Sigma (Taufkirchen, Germany), G7277	1:100 (IG)

## Data Availability

The original contributions presented in the study are included in the article; further inquiries can be directed to the authors.
